# Supplementary material for: Are carbon emissions trading and green financial instruments synergistic? -Comprehensive quantitative research based on content analysis
Source: PLoS One. 2024 Mar 7;19(3):e0298601. doi: 10.1371/journal.pone.0298601 (PMC10919601; doi:10.1371/journal.pone.0298601)
Supplement: S1 Data — (ZIP) [file pone.0298601.s001.zip › Data/地级市Z_F工作报告.zip!/╣π╢1⁄2/╜╥╤⌠╩╨/└┤╫╘╣1⁄2╓┌║┼í╛┬φ┐╦╩2╛▌═°í┐_2019.docx]

各位代表：

现在，我代表市人民政府向大会作工作报告，请予审议，并请市政协委员和其他列席人员提出意见。

2018年工作回顾

过去一年，在省委省政府和市委的坚强领导下，在市人大及其常委会和市政协的监督支持下，我们深入学习习近平新时代中国特色社会主义思想，全面贯彻党的十九大和十九届二中、三中全会精神，对标落实习近平总书记视察广东重要讲话精神和关于广东工作“四个走在全国前列”重要要求，围绕省委“1+1+9”工作部署，按照市委“一四四”工作思路，走正道，出实绩，谋振兴，以“走在前列·百日百项大行动”为抓手，系统化、专业化、精准化推动各项工作取得实实在在的成效。

（一）坚持高质量发展，实现经济运行稳中提质

——经济保持总体平稳。激发微观市场主体活力，把服务企业和项目作为第一要务，精准帮扶企业脱困促产、增资扩产、提质增效，推动经济平稳增长。全年预计实现生产总值2152亿元，同比增长6%左右；规模以上工业增加值630亿元，增长6%；固定资产投资998亿元，增长12%；社会消费品零售总额1122亿元，增长9.5%；外贸进出口总额341.4亿元，下降19.7%；实际利用外资1611万美元，下降4.1%.加大市场主体培育力度，修订市“实体经济十条”，深入开展民营经济政策宣讲和政银企对接，优选表彰30家“梁柱”企业，鼓励支持龙头民营企业扎根发展，新增注册企业8494家，新增“四上”企业355家。

——转型升级加快推进。推进供给侧结构性改革，推动制造业和服务业同步发展，实施重点产业园区建设发展三年行动计划，出台加快服务业发展行动方案。理顺中德金属生态城、揭西电线电缆产业园体制机制，组织实施627个工业技术改造项目，新增省级质检站2家、省级工程技术研究中心8家。优化布局高新区“一区四园”，推动扩容增效升级，启动建设化学与精细化工广东省实验室揭阳分中心，培育新增高新技术企业30家。组织开展“散乱污”企业专项整治，淘汰落后产能企业703家。加快生产性服务业发展，营利性服务业增长66.7%，快递业务量跃居全国第11位，第三产业对经济增长贡献率达到62%.深化产业合作，举办第四届中德（欧）中小企业合作交流会、第十七届中国（揭阳）玉文化节，务实推进与大兴安岭地区对口合作。

——发展效益明显提升。坚持既要有效市场也要有为政府，统筹做好增效益、降成本、促发展、惠民生，切实加强经济管理。全年实现全口径税收154.98亿元，地方一般公共预算收入79.34亿元，增长8.96%，其中税收收入52.06亿元，增长11.73%；为实体经济企业降低成本31.13亿元，其中减免税费20.32亿元；金融机构各项存款余额2273.64亿元、贷款余额1154.75亿元，分别增长7.91%和9.18%；城乡居民人均可支配收入19700元，增长5%；单位生产总值能耗下降3.43%，完成省下达目标任务。

（二）实施区域发展新战略，沿海经济带建设取得重大突破

——现代化基础设施加快建设。揭阳潮汕国际机场旅客吞吐量达到649.4万人次，机场跑道延长及站坪扩建工程启动建设。中石油产品码头和原油码头进展顺利，前詹海上风电专用码头全面复工，大南海公共码头航道工程达到开工条件，神泉示范性渔港不断完善。梅汕客专揭阳段及站场工程加快建设，新建汕汕高铁揭阳段先行开工。揭惠高速建成通车，潮汕环线、汕湛高速、兴汕高速建设有序推进。实施新一轮信息基础设施建设三年行动计划，全市新增光缆里程2800皮长公里，新增光纤接入户18.7万户，光纤入户率提升至92%.开工建设粤东lng配套管线、西气东输三线闽粤支干线等管道工程，推进气化揭阳新能源工程，三江水系连通和半洋隧洞、引韩供水工程全面开工。

——揭阳滨海新区开发工作坚实起步。省政府批复实施《揭阳滨海新区发展总体规划》，构建粤东新城、大南海石化工业区、粤东风电产业园“一中心两基地”产城融合发展新格局，新建汕汕高铁实现与粤港澳大湾区及沿海经济带城市群、产业链串珠成链。争取省政府出台《关于支持揭阳滨海新区粤东新城加快开发建设的若干意见》，联合招商局集团以政企合作新模式启动共建粤东新城。积极开发海上风能，已核准海上风电总装机容量640万千瓦，国电投90万千瓦海上风电率先开工建设，中广核等近海深水区海上风电项目前期工作加快推进，积极引进海上风电研发、风机制造等配套项目，打造海上风电产业基地。

——中石油炼化一体化项目和大南海石化工业区全面启动。聚焦聚力推动和服务中石油项目和大南海石化工业区建设，基本完成各项审批。中委广东石化炼油项目调整优化为年产2000万吨炼油+260万吨芳烃+120万吨乙烯炼化一体化，并纳入国家石化产业规划布局，同步引进建设吉林石化年产60万吨abs、昆仑能源lng等产业链配套项目，总投资达830亿元，项目全面启动。编制大南海石化工业区开发建设规划和产业发展规划，石化大道等主要道路工程基本完工，埔洋村签订搬迁协议，新村主体工程全面封顶，园区配套设施和用地保障加快推进，承载重大项目、吸引重大项目的能力得到增强。

（三）全面加强城市规划建设管理，争创全国文明城市

——城市规划和用地管理不断规范。发挥规划引领作用，揭阳和普宁城市总体规划获省政府批准实施，编制完成揭阳主体功能区规划、滨海新区和惠来总体规划、榕江新城开发单元规划，划定城镇开发边界和生态控制线，组织实施道路建设、乡村规划、全域旅游等一批基础规划，建立规划委员会审查制度，完善城市规划体系。成立土地管理委员会，全面梳理用地管理存在的问题，严格落实以控规单元规范土地征收整备出让工作，加快批而未供、供而未用土地的处置，依法规范推进“三旧”改造，促进节约集约用地。坚决拆违控违治违，处置问题楼盘，加强农村用地管理，无序建设行为得到有效遏制。

——城市基础设施加快推进。开展全域道路改造提升工程，市区揭阳大道、天福东路等“十三路一河一桥”项目全部进入施工阶段，完成国道539线揭东段、省道236线普宁段路面改造工程，升级建设国省道390公里，升级建设县乡道500公里。榕江新城滨江大道、发展大道、潮汕妇女儿童医院、空港一中等一批城市基础设施和公共服务设施加快推进，一批高品质宜居社区集聚建设。全市新建城市小公园82个，新建公共厕所697座，人居环境得到切实改善。新建成6个城镇污水处理厂和172.6公里污水收集管网，新增污水处理规模10.17万吨/日；市区垃圾填埋场二期建成投入使用，市区和普宁两座生活垃圾焚烧发电厂点火试运行，一批县镇级垃圾填埋场整治达标。

——城市管理水平有效提升。以创文统领“五城同创”，深入开展“五河毓秀”“五路绿化”“五水清漂”“五边清污”行动，城乡面貌有了明显改观。开工中小河流整治项目22宗，绿化道路1997公里，榕江等主要河流基本实现无水上漂浮物，一大批卫生死角得到清理。网格化治理城市“六乱”，综合整治交通秩序，规范交通出行，提升道路通行效率。文明创建活动深入开展，社会文明程度不断提高。

（四）聚焦决胜全面建成小康社会，坚决打好打赢“三大攻坚战”

——脱贫攻坚和乡村振兴深入推进。开展162条省定贫困村和相对贫困人口脱贫攻坚，改造农村危房351户，全年新增达到脱贫条件人口2万人，农民人均可支配收入可望达到14400元，增长9%.建设普宁青梅、惠来南药2个省级现代农业产业园，举办梅花节、樱花节、番茄节等特色农业节庆，新增国家地理标志商标1个，新增农业类省名牌产品6个，获评中国美丽休闲乡村1个、“十佳”最美广东农业公园2个。推进耕地保护和高效节约利用，开垦水田3098亩，建设高标准农田11.05万亩，全市化肥农药使用量实现零增长。以农村人居环境整治为重点，扎实推进社会主义新农村建设，开工644个农村雨污分流工程项目，农村保洁实现全覆盖。农村土地确权和粮食生产功能区、重要农产品生产保护区划定工作基本完成，揭东区升级为国家农村集体产权制度改革试点。

——污染防治和生态环境保护攻坚克难。集中力量整治练江流域污染，系统推进污水处理厂及配套管网建设、纺织印染环保综合处理中心建设、开展印染行业污染整治、“1+10”河道综合整治、垃圾收运系统建设、农村农业面源治理等工作，成立练江母亲河保护基金；组织实施榕江水环境改善和东湖、龙石国考断面达标攻坚“1+10”措施，排查整治重点支流（河涌）68条，全面推进污水处理设施建设，大力开展重点工业污染治理和榕江南北河清理保洁；坚持“五河毓秀、枫江先行”，加大力度拆违整治和解决枫江流域水环境问题。强化大气污染防治，重点加强工业源、移动源污染防治，空气质量6项指标全面达标。组织开展重点行业企业用地土壤污染状况调查，全面加强固体废弃物特别是危险废物处置管理。强化生态环境保护考核，压实河长制，实行单一主体监管和多方联合治理相结合的原则，按照“污染在水里、根源在岸上”的思路，雷霆执法，全市查处环保违法案件293宗，取缔非法排污企业128家，清理禁养区禽畜养殖场（户）1.28万家，封堵非法排口120个。认真抓好中央和省环保督察、国家海洋督察问题整改，设立“曝光台”“回音壁”，推进生态文明共建共治共享。

——风险防控和社会治理稳健有序。清理核实并分类化解地方政府性债务和隐性债务，规范政府举债行为，盘活用好政府债券资金，全市政府债务风险总体稳健可控。统筹推进农合机构改革与风险化解，多措并举压减农合机构不良贷款25.6亿元，支持上市公司化解股权质押和流动性风险，地方金融秩序总体保持稳定。加强食品药品安全监管和卫生防疫工作，有效防控登革热等传染性疾病，严防流入和使用不合格疫苗，严控非洲猪瘟，实现省卫生县城全覆盖。梳理健全“三防”工作体系，有效应对“山竹”等台风和强降水天气。启动道路客运体制改革，实施村级工业区消防安全综合治理试点，安全生产形势持续稳定好转。深入开展扫黑除恶专项斗争，纵深推进“众剑行动”，破获涉黑恶案件557宗，惠来涉毒整治取得阶段性成效。

（五）办好省市民生实事，加强保障和改善民生

——民生保障水平稳步提高。全市财政民生类支出253.3亿元，增长9.9%，占一般公共预算支出的80.7%.支持创业带动就业，实施“潮菜师傅”等职业培育工程，全年城镇新增就业3.2万人。社会保险五大险种新增扩面28.23万人次，企业和居民养老金人均待遇标准分别提高4.25%和23.3%.落实医保支付方式改革，实施医疗、生育保险基金市级统筹，全面取消公立医院耗材加成。取消困难群众住院报销起付标准，住院报销比例较一般标准提高10%左右；大病报销起付标准降至2000元，报销比例提高至80%；门诊单次和累计报销限额增加1倍。基本完成城镇棚户区改造620套，完成市区公租房分配入住3339户。建立健全惠及13万80岁以上高龄老年人生活保障制度，全面完成寺庙收留366名孤儿接收安置工作。

——各项社会事业全面发展。启动创建区域教育中心，积极推进与武汉理工大学合作办学，支持揭阳职业技术学院改革发展，新（改、扩）建中小学校509所、普惠性幼儿园115所，揭东区率先创建成为广东省推进教育现代化先进区。大力实施文化惠民工程，新建300个基层综合性文化服务中心示范点、20个乡镇（街道）文体广场，新增省级非物质文化遗产7项，“三山国王”祖庙成为省海峡两岸交流基地。深入开展“贤德揭阳、揭阳进贤”等主题群众文化活动，社会主义核心价值观广为弘扬。制定实施《“健康揭阳2030”行动计划》，推进建设360个医疗卫生机构基础设施项目，市残疾人康复和托养中心基本完工，新建成10个残疾人社区康园中心，人均公共卫生服务经费提高至每年55元。

此外，第四次经济普查、第二次污染源普查有序推进，信访、档案、史志、气象、地震、外事侨务、民族宗教、新闻出版、广播电视、人民防空、国防动员、海防打私、民兵预备役、双拥、红十字会、社科、文联等工作也取得新进展。

（六）突出履职尽责，着力提升政府治理能力和治理水平

——坚持党的领导。实现党对政府工作的领导制度化，持续深入学习贯彻习近平总书记重要讲话和指示批示精神，规范和加强市政府党组工作，完善“三重一大”决策机制，政府谋大事、抓落实、促发展的能力明显增强。全面落实党风廉政责任制，严格执行中央八项规定精神，持之以恒正风肃纪，营造风清气正的政务环境。

——坚持依法履职。建立重大决策出台前向人大常委会报告制度，自觉接受市人大、市政协及各界监督，定期向市人大及其常委会报告工作，向市政协通报情况，人大代表建议和政协委员提案办结率及满意率均达到100%.坚持依法行政，深入学法用法，定期听取行政执法情况汇报，推动建立行政执法与刑事司法相衔接的工作机制。深入开展“怕慢假庸散”专项整治，大力倡导“严、实、细”工作作风和“系统化、专业化、精准化”工作方式，着力做好基础性、技术性工作。

——坚持改革创新。落实“1+1+10+n”改革总体布局，制定实施揭阳滨海新区创建新时代改革创新先行区等一批改革行动方案。开展财政预算体制改革，探索建立以支出项目和支出科目统筹所有财政资金的分地区、分行业、保重点的财政工作体系。加强政府投资项目管理，严格落实基本建设程序，开展建设项目审批制度改革，稳步推进代建制。深化“放管服”改革，取消行政审批事项30项，清理规范各类行政职权中介服务事项118项，推进政务数据系统对接、数据共享和服务事项“智能办”“指尖办”。按照中央和省统一部署推进政府机构改革，市级机构改革方案获省批准实施。

以上这些成绩的取得，是习近平新时代中国特色社会主义思想光辉引领的结果，是党中央国务院、省委省政府和市委坚强领导的结果，是市人大、市政协和社会各界监督支持的结果，是全市上下团结奋进、努力拼搏的结果。在此，我谨代表市人民政府，向全市干部群众、企事业单位、驻军官兵、各民主党派和人民团体，向关心支持揭阳发展的港澳台同胞、海外侨胞、国际友人和各界朋友致谢致敬！

同时，我们也清醒地看到，我市经济社会发展和政府自身建设仍存在短板和不足：一是经济运行稳中有忧，下行压力较大，工业转型阵痛加剧，城市经济综合效应尚未完全释放，新产业新动能还不足以形成有力支撑，转型升级、创新驱动、高质量发展十分紧迫；二是生态环保、文明创建尚未取得决定性、压倒性胜利，问题和矛盾更多集中到基础设施配套建设和体制机制建设上；三是教育、文化、卫生、体育等民生领域供给质量有待提高，脱贫攻坚、新农村建设还需加大力度；四是改革开放还存在不彻底、不到位的地方，营商环境还需要进一步改善，思想要再解放、改革要再深入、工作要再抓实；五是有效市场与有为政府协同作用有待加强，特别是政府管理经济、推动高质量发展的能力仍需进一步提高。对此，我们将认真研究，并采取切实有效措施加以解决。

2019年工作安排

2019年是新中国成立70周年，是全面建成小康社会的关键之年，也是我市全面建设广东沿海经济带新增长极的开局之年，做好今年各项工作至关重要。我们要见“著”知“微”，坚持从长期大势认识当前形势，从全国、全省的发展战略中找动力，把握好国家强化逆周期调控和促进形成强大国内市场等宏观政策“窗口期”，系统谋划，专业设计，精准施策，加大投资补短板，提升供给促消费，以更实的作风、更实的举措推进各项工作，增强揭阳发展内生动力，更好满足人民群众对美好生活的向往。

今年政府工作的总体要求是：高举习近平新时代中国特色社会主义思想伟大旗帜，深入学习贯彻习近平总书记视察广东重要讲话和指示批示精神，围绕“五位一体”总体布局和“四个全面”战略布局，认真贯彻落实中央经济工作会议和省委十二届六次全会、市委六届六次全会精神，坚持稳中求进工作总基调，坚持以新发展理念和高质量发展要求为统揽，以供给侧结构性改革为主线，贯彻“巩固、增强、提升、畅通”八字方针，坚持“一四四”工作思路和百日百项大行动，走正道，出实绩，谋振兴，真落实，努力把揭阳建设成为广东沿海经济带新增长极。

今年经济社会发展的主要预期目标是：生产总值增长7%左右；规模以上工业增加值增长6.8%；固定资产投资增长12%；社会消费品零售总额增长9.5%；外贸进出口总额增长3%；实际利用外资增长10%；地方一般公共财政预算收入增长7%以上；城乡居民人均可支配收入增长7%；居民消费价格涨幅控制在3%以内；城镇登记失业率控制在3.5%以内；节能减排约束性指标完成省下达年度计划。

实现上述目标，必须围绕以下十个方面精准发力：

（一）围绕建设“一轴一带一区”精准发力

服务中石油项目大建设催生大平台。把中石油炼化一体化项目作为“一号项目”，举全市之力支持好、服务好，抓紧完成环评、用海用地报批、主体工程和库区及管线工程的征地拆迁等工作，加快炼油部分投资建设进度并开工建设化工部分，掀起投资建设大会战。依托中石油炼化一体化项目，推进吉林石化年产60万吨abs、昆仑能源揭阳lng等产业链配套项目落地同步建设。围绕服务“一号项目”打造“一号平台”，完善优化大南海石化工业区规划布局，以服务中石油炼化一体化项目为核心，开工建设周边道路、雨水明渠、污水处理、固废处理以及供电、供水、通讯等重要配套设施，确保同步建设、同步投入使用，统筹抓好园区土地征收和集中管理，提升园区管理服务能力，加快石化中下游产业链布局和招商选资，建设绿色、智能、效益型世界级石化基地。

支持惠来打造沿海经济带主战场。以重大项目、重点平台和重要基础设施建设为龙头，推动港产城融合发展。加快风电产业园配套道路、供电、供水和污水处理厂网等重点基础设施建设。全力推进国电投海上风电暨前詹专用码头建设，争取开工近海深水区海上风电项目，精准引进风机制造、海工装备、船舶安装、水下工程、冷能利用等产业链配套项目，打造海上风电产业和运维基地。深化粤东新城政企合作模式，用好省专项扶持政策，落实开发规划和开发条件，推动起步区开工建设。围绕对接风电产业园、大南海石化工业区和粤东新城抓好惠来县城扩容提质，重点抓好联动性、带动性道路基础设施建设，优化提升民生和公共服务设施建设水平和服务能力。

促进中心城区一体化发展。中心城区既要树立“大市区”理念，也要突出核心区功能，要注重组团集聚，切忌摊大饼式发展。榕城区要顺应古城、新城发展规律，加强“古城文化旅游”规划设计，理顺公有房屋管理体制，做好古城整体保护开发和旅游经营，以旅游开发提升古城整体形象；要聚焦建成区低效用地盘活整备和问题楼盘处置，更加注重品质，加快打造揭阳大道城市中轴线，加快城市“优二进三”，大力发展现代服务业。揭东区要坚守工业阵地，抓好揭东经济开发区、中德金属生态城两个价值创新园区建设运营；围绕融入和服务核心区，强化基础设施和公共服务配套。揭阳产业园要依托省道234线抓好园区经济和城区经济，以土地高效利用为基础，抓好产业招商，以项目为龙头，实现“一条扁担挑两头”。空港经济区要以交通道路建设和土地征收整备为重要抓手，以项目引进和建设为龙头，加快榕江新城开发建设；大力培育发展临空产业，优化调整快递物流布局，推动城市品质化、产业高端化。

推动各功能区板块组团发展。揭普（潮）惠发展轴要围绕揭阳大道—揭惠高速，加快交通基础设施互联互通，集聚优质资源，促进城市开发建设和产业优化布局，推动揭阳主中心与普宁、惠来次中心串珠成链，实现海陆空功能联动。普宁要大力发展现代商业、生产性服务业和高端制造业；加快英歌山等产业园区建设，推动传统产业转型升级；推进城市成片开发、扩容提质；把生态环保工作摆在更加重要的位置，着力抓好污水处理厂、管网等环保设施建设，以及纺织印染企业入园和河涌治理，全面推进练江流域综合整治；加快推进国道238线升级改造和高速公路连接线工程，更好与惠来滨海新区实现联动发展。西部生态发展区以揭西为主体，要把保护和修复生态、提供生态产品作为首要任务，因地制宜发展休闲农业、文化旅游、养老养生等生态产业，同时推进县城、镇域和乡村一体化建设，抓好脱贫攻坚和乡村振兴工作。

（二）围绕发展实体经济精准发力

支持民营企业发展壮大。推进市场主体培育工程，实行差别化服务措施，支持龙头企业做强做大，带动中小企业转型升级，培育小微企业上规模、上档次。帮助企业依法依规理顺历史用地手续盘活资产，提高工业用地容积率，建设工业服务基地或工业大厦，支持符合条件的企业实施“三旧”改造。加强政银企对接合作，推动金融资源更多投向实体经济。支持企业组团开拓国际国内市场，鼓励企业进口先进设备和开展对外合作。大力培育和弘扬企业家精神，把投资有回报、产品有市场、企业有利润、员工有收入、政府有税收、环境有改善作为衡量发展质量和效益的标准。

加强产业园区建设管理。按照“项目+园区+产业”的工作思路，每个县（市、区）集中力量服务1个龙头产业项目，打造1个重点产业园区，培育1个主导产业。严格管好产业用地，提高产出效益，按照在建、批而未供、供而未建、可供可招商、可征可报批的量化任务，分类梳理园区工业用地，对应制定跟踪服务计划、开工建设计划、用地收回计划、招商供地计划、征地报批计划，精准推动项目落地建设；盘活园区现有物业和厂房资源，实行“零地”招商；支持企业统筹使用城区的存量工业用地和园区的新增工业用地，走“研发+展销+生产基地”的集约节约用地新路子。强化政府主导作用和园区管委会主体责任，实现“统一规划、统一征收、统一建设、统一招商、统一管理”，市将制定下达产业园区基础设施建设计划并实行考核，完善道路、供电、供水、通信、环保等园区基础配套。探索政企合作开发园区新模式，支持行业协会和大型企业建设集约化生产基地，组织企业入园集聚和提供产业服务配套，标准厂房可以分栋分层确权，鼓励以厂房租赁等方式推动工业轻资本运营。着力理顺园区管委会与所在乡镇（街道）的经济社会管理权责划分和统辖关系，协同做好园区管理服务。

推进招商引资和转型升级。各地要紧紧依托产业园区，在做好土地资源整理规划基础上，按照高质量发展要求，结合自身定位和资源条件，精准招商，开放招商，着力引进精细化工、海工装备、临空制造、新能源、新材料、人工智能、节能环保、生物医药等先进制造业，以及与产业链配套相关的研发设计、现代金融、信息服务等生产性服务业，同时积极引导传统产业入园集聚、转型升级。今年要下达招商引资目标任务并实行量化考核，以项目落地为依据实施专项督查，每季度检查一批在建项目并推动一批项目开工或签约。推进实施新一轮技术改造三年行动计划，重点支持企业运用数字化、信息化手段更新设备、改进工艺、提升技术。着力建设好榕江实验室，做实现有科技创新公共平台和企业研发机构，加大高新技术企业培育力度。加快申报创建国家级高新区，促进全市高新技术产业集聚。以市场化方式运营好科技孵化器、众创空间等“双创”平台，支持发展电子商务、创意设计、“互联网+”等新业态，培育经济新动能。

（三）围绕提升城市建设管理运营水平精准发力

持续加大城市资源管控力度。开展第三次国土调查，强化规划引领，编制国土空间规划，科学管好用好每一寸土地。开展土地管理专项行动，抓好控规单元、征收整备方案、科学分配和安排用地指标、资金保障、用地报批、招商与规划出让条件、土地现场管护和批后监管等环节，落实控规单元规范土地征收整合储备，结合“三旧”改造盘活土地资源，储备城市建设用地，有序服务项目招商和城市发展。加大用地促建、闲置土地清理、低效用地盘活和违法建设治理，提升土地资源使用效益。

聚焦推进榕江新城开发建设。集中资源把榕江新城建设成为科技新城、产业新城、服务新城，打造现代化城市。重点抓好望江北路、滨江大道、进贤门大桥、京灶大桥等大区域循环交通要道建设，谋划启动新一批道路工程，形成交通、景观和城市发展引领功能；加大城市开发经营力度，推动建设一批医院、学校、产业园区、楼宇服务、高品质宜居社区等城市功能项目，形成新的城市发展动能和满足群众需要的新区新城；抓紧添置和配置公园、绿地、环卫设施、文化设施、交通设施及路标路牌等一批“城市家具”，不断提升新城品质。

以服务业培育壮大城市经济。组织实施《揭阳市2018-2020年加快服务业发展行动方案》，围绕服务业八大类细化措施，加快平台载体建设、市场主体培育和具体项目落地。顺应城市发展规律，加强项目规划策划，积极引进建设城市综合体、酒店、高端商务区、科技服务等配套项目，增加商品房市场投放量，促进房地产市场提质增效。依托机场、港口、高铁、高速等现代化基础设施，优化快递物流产业布局，吸引龙头企业落户集聚，机场片区重点发展临空型高端物流，港口片区重点发展远洋重型物流，高铁片区重点发展时效型中高端物流，高速沿线重点发展普通陆路物流。

建立健全城市管理长效机制。以“创文”为抓手，通过持续不断的“专项行动+机制建设”常态化工作，构建“村居为基础，镇街为主体，区级抓统筹，市级保重点”的城市管理运行体系。推动建立“镇街+村居+城管+公安”联合执法机制，细化明确城市管理事权责任，寓管理于服务之中，常态化抓好“六乱”整治、环卫保洁、建筑工地、集贸市场、交通秩序、公共服务设施、绿化景观等城市管理工作。从规划用地、报建报批、销售管理、竣工验收、不动产登记等环节入手，全面理顺房地产开发管理体系，从源头上解决问题楼盘和违法建设，促进房地产市场健康发展。

（四）围绕建设现代化交通体系精准发力

加快现代化交通基础设施建设。完成揭阳潮汕国际机场跑道延长及站坪扩建工程，启动机场航站楼二期扩建和交通换乘中心建设工程，争取开辟更多国际国内航线，提升机场运载能力，完善机场总体规划，为建设第二跑道和打造交通便利的枢纽中心留足空间。推进前詹专用码头建设，争取大南海公共码头开工建设，系统谋划136公里海岸线的港口码头和临港产业布局，打开走向海洋经济的“大门”。服务好梅汕客专、汕汕高铁主线建设，加快推进疏港铁路规划建设，支持推进铁路土地综合开发。加快潮汕环线、汕湛高速、兴汕高速建设。建立政企联动工作机制，系统抓好电力、通信、油气管道和综合管廊等规划建设，重点推进西气东输、粤东lng、昆仑燃气等天然气管线项目建设。

完善城乡互联互通体系。基本建成市区“十三路一河一桥”，启动实施新一批路桥修补加固工程，打通一批断头路、中梗路，开工建设望江北路空港段、206国道改线揭东段等一批重点市政道路工程。同步推进揭惠高速市区连接线、新芦连接线、麒麟互通和潮汕环线京灶大桥连接线等建设，着力解决高速公路与市区、城区、镇区的快速联系，同时实施25个高速路口综合整治和7个未通车高速出入口连通接顺工程，提升高速公路运输效率和交通形象。推动惠来安澜桥至神泉镇区等一批县乡道升级建设为国省道，组织实施“四好农村公路”建设计划，加快形成四通八达的公路网络。

加强城市交通管理。对市区路口交通设施实施微改造，分批推进路口渠化和完善交通配套，着力解决建设与管理、设施与技术不同步、不配套问题。完善城区交通信号系统、路牌指引、路面标识，提升车辆通行效率与行车安全。理顺国有交通运输企业产权体制，重组交通运输主体，科学优化客运站点规划布局，加强行业管理，规范运输市场秩序。大力发展城市公交，优化市区公交线路和站点布局，扩大公共交通城乡覆盖范围，改革公交补助机制，方便群众出行。编制市区停车场所建设专项规划，将现状空地改造成“小公园+停车场”“小绿地+停车场”，增加公共停车位，支持一体化建设人防设施和地下停车场，鼓励建设经营性停车场，缓解城市停车难题。严格交通执法，加强社区警务管理和农村道路“两员两站”建设，倡导文明出行。

（五）围绕乡村振兴精准发力

全力推进脱贫攻坚。发挥好“产业扶贫资金+社会资本+土地流转”的作用，依托特色农业和特色农产品，引进龙头企业，发展规模经营，实现资产收益扶贫、产业带动脱贫。坚持扶贫与扶智、扶志相结合，帮助扶贫对象提升技能、转移就业、稳定就业，形成可持续的收入来源。完善建档立卡动态管理机制，加强跟踪服务，确保“两不愁三保障”落实到位，强化“兜底”脱贫，切实减少和防止脱贫人口返贫。

加快农村产业构建。重点抓好普宁青梅、惠来南药、揭西茶叶、揭东竹笋、惠来凤梨、普宁青榄等6个现代农业产业园提质发展，组织实施十大现代农业产业园、十大农鲜特产品等现代农业“八个十”建设，创建一批省级“一村一品、一镇一业”富民兴村产业示范镇村。加强乡村旅游资源整体开发和品牌打造，支持举办各种特色农业节庆，大力发展农家乐、渔家乐、林家乐等乡村旅游项目。用好革命老区红色资源、政策资源，加大老区开发建设力度，因地制宜发展红色旅游。加快培育家庭农场、农业合作社等新型农业经营主体，推广“龙头企业+合作社+农户”经营管理方式，推动农业规模化经营，鼓励产业链各环节通过股份合作实现利益联结、农工商多向多元合作，带动农民持续增收、走向富裕。落实粮食安全和“菜篮子”负责制，推进生猪屠宰管理，加强非洲猪瘟等动植物疫病防控，保障农产品充分和安全供给。

加快新农村建设。突出保留乡土气息，抓好规划设计，确定整治创建内容，以规划引领统筹项目和资金安排，分步打造社会主义新农村。以村容整洁为重点，以“三清三拆”为基础，推进雨污分流、垃圾清运、水岸同治、“厕所革命”、绿满家园、村道硬底化、自来水到户、光纤到户等农村基础设施建设。乡镇要抓统筹管理，村居要发挥主体作用，抓好每个村一张规划图和一张建设项目清单，根据筹集资金确定实施项目，坚持公开、简便、务实、高效，提升财政专项资金使用效率，加快生态宜居美丽乡村建设。

加强乡村治理和乡风建设。加强基层组织建设，落实基层活动阵地，建立健全村（居）干部工资保障机制，切实发挥村支部战斗堡垒作用。规范村级组织议事决策程序，发挥乡贤咨询委员会作用，形成民事民议、民事民办、民事民管的基层协商格局。推进农村集体产权制度改革，规范农村集体“三资”管理，建立健全集体产权流转交易和收益分配机制。加强农村法治宣传教育和法律服务，深入推进“一村一法律顾问”工作，引导农民依法表达诉求、解决纠纷、维护权益。推动殡葬改革等移风易俗，引导农民弘扬新时代新风。

（六）围绕推进生态文明建设精准发力

强化工程截污。围绕榕江、练江、枫江三江达标治理，以充分发挥污水处理设施作用为核心，统筹三江流域污水处理厂网一体化建设，实现镇级污水处理厂和农村雨污分流工程全覆盖，新建污水管网150公里以上。积极对接省属国企参与三江流域环保设施建设运营，理顺建设运营管理体制。全面建成普宁垃圾焚烧发电厂、市区绿源垃圾综合处理与资源利用厂，确保设施配套、运营高效。抓好普宁纺织印染环保综合处理中心、中德金属生态城电镀园全行业整合入园，强化集中治污。加快石化环保中心建设，提升危废处置能力。

强化源头管控。改革生态环境保护管理体制，深化中央和省环保督察及“回头看”反馈问题整改，系统推进水、大气、土壤、固体废物、农业农村污染整治行动计划。全面落实单一主体监管和多方联动治理相结合的工作机制，压实河长和属地管理责任，集中开展“五清”专项行动，加强水质监测和污染物成因分析，“一河一策”推进重点河涌和黑臭水体综合整治，实现水质达标。推进污染源普查和动态管理，从严整治“散乱污”企业和工业、农业面源污染，严厉打击非法转移倾倒固废行为。加强建筑工地扬尘管理，加大燃煤、锅炉、露天焚烧、烧烤排查治理，大力整治交通运输重点车辆遗漏飘洒，鼓励老旧车淘汰。建立土壤污染重点管控清单，及时开展污染源头管理，严防土地污染。

强化生态修复。全力推进“三江连通”、半洋隧洞引水工程、引韩供水工程“三大水系”工程建设，谋划实施新一批中小河流治理工程，进一步提升水生态、水景观。加强耕地保护，严格土地矿产卫片执法，积极推进高标准农田、水田垦造和农村拆旧复垦。加强海域海岛巡查保护，严格管控围填海，推进自然岸线修复和美丽海湾建设。巩固深化“五路绿化”成果，启动建设5000亩沿海基干林带和万里碧道示范段，加强自然保护区管理，抓好黄岐山、紫峰山、桑浦山等森林公园建设，支持揭西县争创“中国天然氧吧”品牌，全力创建国家森林城市。

（七）围绕推进民生事业发展更平衡更协调精准发力

扎实办好教育事业。围绕建设区域教育中心，统筹抓好高等教育、基础教育和职业教育改革发展，实现创建广东省推进教育现代化先进市目标。从师资队伍、专业建设、基础设施等方面支持揭阳学院创建高水平有特色高职学院；推进与武汉理工大学合作办学，加快在粤东新城创办高等院校。加大专项投入，提升市直和城区学校办学水平，推进农村学校“全面改薄”和信息化改造，推动一批普通高中学校创省一级学校。落实学前教育三年行动计划，统筹推进公办、普惠性民办幼儿园建设，提高优质学前教育资源供给。调整优化中职教育资源布局，强化“双精准”育人，开工新建1所中职学校。启动建设教师发展中心，培树“贤德教师”，积极稳妥推进中小学教师“县管校聘”管理改革，规范课后托管并加强监管。

加强医疗卫生服务。整合中心城区公共卫生资源，调整优化市区医疗服务布局，积极推动区级人民医院升级建设市级专科医院。推进5家市级医疗卫生机构、11家县级医院、5家中心卫生院、39家乡镇卫生院升级改造和标准化建设，抓好普宁、揭西、惠来三个急救体系和紧密型医联体建设，提升各级医疗卫生服务能力。推进规范化村卫生站全覆盖和家庭医生签约服务扩面提质，加强基本公共卫生服务，筑牢老百姓身边的第一道健康防线。推进“三医联动”改革，落实公立医院薪酬制度改革，完善医保支付制度设计，全面推行药品集团采购，建立健全分级诊疗体系。强化各层次人才配备，落实基层医疗人员县招县管镇用制度，引导优质人才资源下沉基层，切实形成服务能力。大力传承和保护中医药文化，完善中医药服务体系，支持发展康养产业。加强公共卫生服务，切实抓好“除四害”、蚊媒监测和季节性流行病预防控制等工作，扎实创建国家卫生城市。

发展文化体育事业。加强公共文体设施建设，完善市文化中心功能配套，开工建设市体育学校，推进建设一批镇村文体设施示范点，落实文化惠民、全民健身。启动中山路等骑楼特色街立面修复和南北滘河等水乡景观修复，推进丁氏光禄公祠等重点文物保护单位修缮，抓好文化产业基地（园区）、“非遗”传承保护基地等平台建设，继续举办玉文化节等特色节会，推动文化旅游融合发展。持续组织开展“贤德揭阳、揭阳进贤”等群体性文化活动、文化志愿者服务活动，大力弘扬社会主义核心价值观，促进城市精神文明建设。

提升就业创业和社会保障水平。实施就业优先战略，加强就业公共服务，广泛开展职业技能培训，推动高校毕业生、农村劳动力、异地务工人员、退役军人等重点群体多渠道就业创业，稳妥做好军转干部安置工作。落实好人才政策，加大人才创新创业激励力度，优先解决高层次人才出入境与居留、配偶安置、子女教育、落户、住房、医疗、金融服务等问题，打造“最优人才发展生态圈”，吸引更多海内外优秀人才来揭阳创新创业。围绕企业养老保险省级统筹抓好扩面征缴，健全待遇正常调整机制，稳步提高各类社保待遇水平。强化民生“兜底”，抓好市残疾人康复和托养中心、儿童福利院、救助管理站、救助安置中心等项目建设运营，完善城乡低保、医疗救助、临时救助等社会救助体系。

办好民生十件实事。一是打通一批断头路，改造一批破损路，形成一批循环路，推动每个乡镇（街道）至少改造1条社区道路，解决路不通、路不平、路不畅等问题。二是加强城市现状空地和边角地、插花地整治，建设50个以上“小公园+停车场”“小绿地+停车场”或城市景观小品。三是综合整治练江“1+10”流域河道和榕江68条重点支流（河涌），完成全市288个自然村的雨污分流工程建设项目。四是新（改、扩）建43所中小学校，并将公办幼儿园和普惠性民办幼儿园覆盖率提高到75%以上。五是新开工519家村卫生站规范化建设项目，并将村卫生站基本医疗纳入医保门诊报销。六是建设揭阳潮汕星河青少年活动中心，新建16个乡镇（街道）文体广场和30个体育场地设施示范点。七是为城乡居民基本养老保险和基本医疗保险等公共服务开通移动支付、银行转账等自助缴费方式。八是推进城镇棚户区改造，基本建成住房1260套。九是实施15个以上集贸市场标准化综合改造示范工程。十是推进“厕所革命”，新（改、扩）建公共厕所100座，确保市区主次干道、车站和集贸市场公厕不低于二类标准。

同时，组织推进第四次经济普查、第二次污染源普查，做好地方志、新闻出版、广播电视、外事、侨务、台港澳、军民融合、国防动员、民兵预备役、双拥、人防、气象、地震、民族宗教、妇女儿童、红十字会、社科等各项工作。

（八）围绕营造共建共治共享社会治理格局精准发力

着力防范化解重大风险。加强政府债务管理，在规范政府投资项目和举债行为的前提下，把握国家积极财政政策加力提效，较大幅度增加地方政府专项债券规模的契机，积极谋划好项目，提高债券资金使用效益，支持政策性、开发性金融深度参与城乡基础设施和公共服务设施建设。统筹推进农合机构改制和风险化解，运用法治手段清收欠账和处置股权，大力打击恶意逃废银行债务、非法集资和互联网金融诈骗等犯罪行为，规范地区金融秩序。指导上市企业对接用好国家和省扶持政策，积极化解股权质押和流动性风险，促进企业持续健康发展。加快城投公司、水务集团整合组建步伐，做实国有资产和经营主业，妥善处置国有“僵尸企业”历史遗留问题，推动国有经济健康发展。

加强社会治安综合治理。坚决落实意识形态责任制，维护政治安全。把扫黑除恶专项斗争同“众剑行动”、行业治理、基层治理、部门监管结合起来，突出镇村工作重点，重拳打击乡匪村霸、非法占用土地、非法买卖土地、盗采资源、污染环境、工程围标串标和垄断建筑物料等影响公平正义、破坏生态环境、“微腐败”黑恶势力，深入开展涉毒专项整治，严厉打击假币、黄赌毒黑拐骗、“两抢一盗”、电信网络诈骗、网络传销等各类犯罪，以智慧新警务建设为抓手提升综合治理信息化水平，努力营造良好的社会环境。

建立健全应急管理体系。全面落实安全生产责任，持续抓好生产安全、工程安全、交通安全、水上安全、景区安全、消防安全、森林防火等领域隐患排查整治。推进“大应急”指挥体系建设，建立系统完备、快速高效的指挥、信息、责任、队伍和应急处置工作体制机制，强化应急处突主动权，最大程度减少灾害和事故损失，保障人民群众生命财产安全和社会稳定。

（九）围绕新时代改革开放再出发精准发力

深化体制机制改革。落实市县机构改革方案，有序做好职能和人员划转，科学设计内设机构和职能配置，确保每项工作都有人抓、有人管、有人负责。推进镇街行政管理体制改革和基层财政保障机制改革，确保责、权、利统一。抓好综合行政执法改革，着力理顺职能部门与派出机构的关系，完善综合行政执法统筹机制。适应和对接省级部门“大专项+任务清单”预算制度改革，理顺市直部门财政预算执行关系和市县区财政管理关系，强化财政预算的编制、执行、监督、管理等工作，明确预算管理权责，构建大财政大预算格局，以项目统筹资金，推动预算管理提质增效。改革政府投资市属非经营性项目管理，稳妥推进实施代建制，切实提高项目建设效率和资金使用效益。

优化营商环境。统筹推进“放管服”与数字政府建设，优化审批流程，加强数据共享，围绕少填、少报、少走、快办推进减证便民，努力让老百姓办事“最多只跑一趟”。更大力度推进商事制度改革和建设项目审批制度改革，全面落实降税减费政策，切实降低企业制度性交易成本。坚持市场化改革方向，推动国企改革从管企业向管资本转变，全面清理整治中介服务市场，加强公共资源交易市场管理，坚决扫除影响市场发挥决定性作用的体制机制障碍。完善市场监管体系，强化产品质量和食品药品安全监管，营造公平开放、竞争有序的市场环境。

扩大开放合作。积极参与“一带一路”沿线国家经贸合作，继续承办中德（欧）中小企业合作交流会，发挥驻德中国中小企业中心等平台作用，争取更多合作项目落地。强化湾区意识、湾区行动，在发展战略、交通基础设施、产业布局、制度设计上，与粤港澳大湾区加强对接，更好参与广东沿海经济带和“一带一路”建设。按照“潮人北上、山货南下”的总体思路，推动揭阳庞大的消费市场资源与大兴安岭丰富的生态旅游、绿色农产品资源深度对接，创造更多务实成效。

（十）围绕政府治理能力和治理体系现代化精准发力

推进法治政府建设。全面加强党的领导，持续深入学习贯彻习近平新时代中国特色社会主义思想，强化政治引领，增强“四个意识”，坚定“四个自信”，坚决做到“两个维护”。牢固树立宪法意识，严格按照法定权限和程序行使职权、履行职责，完善政府法律顾问和科学民主决策机制，建立健全行政执法与刑事司法相衔接的工作机制。扩大政务公开，自觉接受市人大、政协和社会监督，完善政府与人大代表、政协委员联系沟通机制，认真办理人大代表建议和政协委员提案。支持人民法院依法受理行政案件，及时化解行政争议，做好矛盾纠纷排查，加强信访工作，维护社会和谐稳定。

推进有为政府建设。大力弘扬新时代揭阳走在前列精神，全面践行“系统化、专业化、精准化”工作要求，培育打造忠诚干净担当和懂政策懂业务的干部队伍。着力提升政府赋能市场、管理经济的能力和水平，改进经济运行监测分析调度方式，推动优质资源向优质产业和优质企业集中，促进经济高质量发展。强化政府主导项目建设职责，严格落实基本建设程序，切实加强对设计、造价、施工等关键环节的管理，确保项目实施过程规范、透明、优质、节约。

推进清廉政府建设。加强政府系统党的建设，深入落实中央八项规定精神及省市实施办法，持之以恒整治清除“四风”问题，特别是官僚主义、形式主义。适应监察体制改革，转变行政监督方式，加大审计监督和督查问责力度，着力增强政府的执行力和公信力。建立健全政府廉洁风险防控体系，重点从制度建设入手，开“前门”，堵“后门”，完善公务员正向激励机制，营造风清气正的良好政治生态。

同志们！梦想是拼出来的！让我们更加紧密团结在以习近平同志为核心的党中央周围，高举习近平新时代中国特色社会主义思想伟大旗帜，深入学习贯彻习近平总书记重要讲话和批示指示精神，全面落实省委十二届六次全会、市委六届六次全会精神，走正道，出实绩，谋振兴，真落实，一年接着一年干，一站接着一站跑，以优异成绩庆祝中华人民共和国成立70周年！
